# Fusion of dual modalities of non-invasive sensory feedback for object profiling with prosthetic hands

**DOI:** 10.3389/fnbot.2023.1298176

**Published:** 2023-12-13

**Authors:** Jie Zhang, Chih-Hong Chou, Manzhao Hao, Yan Li, Yashuo Yu, Ning Lan

**Affiliations:** ^1^Laboratory of NeuroRehabilitation Engineering, School of Biomedical Engineering, Shanghai Jiao Tong University, Shanghai, China; ^2^Institute of Medical Robotics, School of Biomedical Engineering, Shanghai Jiao Tong University, Shanghai, China; ^3^Ruijin Hospital, Shanghai Jiao Tong University School of Medicine, Shanghai, China

**Keywords:** somatotopic sensory feedback, evoked tactile sensation, substitute sensory feedback, sensory fusion, transcutaneous electrical nerve stimulation

## Abstract

**Introduction:**

Either non-invasive somatotopic or substitute sensory feedback is capable of conveying a single modality of sensory information from prosthetic hands to amputees. However, the neurocognitive ability of amputees to integrate multi-modality sensory information for functional discrimination is unclear. The purpose of this study was to assess the fusion of non-invasive somatotopic tactile and substitute aperture feedbacks for profile perception of multiple physical features during grasping objects.

**Methods:**

Two left transradial amputees with somatotopic evoked tactile sensation (ETS) of five fingers participated in the study. The tactile information of prosthetic hand was provided to amputees by the ETS feedback elicited on the stump projected finger map. Hand aperture information was conveyed to amputees with substitute electrotactile stimulation on the forearm or upper arm. Two types of sensory feedback were integrated to a commercial prosthetic hand. The efficacy of somatotopic ETS feedback on object length identification task was assessed with or without substitute aperture stimulation. The object size identification task was utilized to assess how ETS stimulation at the stump may affect aperture perception with stimulation on the ipsilateral upper arm or forearm. Finally, the task of identifying combined length and size was conducted to evaluate the ability of amputees to integrate the dual modalities of sensory feedback for perceiving profile features.

**Results:**

The study revealed that amputee subjects can effectively integrate the ETS feedback with electrotactile substitutive feedback for object profile discrimination. Specifically, ETS was robust to provide object length information with electrotactile stimulation at either the forearm or upper arm. However, electrotactile stimulation at the upper arm for aperture perception was less susceptible to the interference of ETS stimulation than at the forearm.

**Discussion:**

Amputee subjects are able to combine somatotopic ETS and aperture feedbacks for identifying multi-dimensional features in object profiling. The two sensory streams of information can be fused effectively without mutual interference for functional discrimination.

## 1 Introduction

The fusion of proprioceptive receptors and cutaneous mechanoreceptors contributes to feeling and manipulating the external object (Edin and Abbs, 1991; Edin and Johansson, 1995; Kandel, 1999). Restoring tactile and proprioceptive information resulting from the loss of hand shows its improvement for the daily life of forearm amputees (Marasco et al., 2021; Vargas et al., 2021; Rostamian et al., 2022). Neural tactile afferents from hand digits provide necessary information about spatiotemporal patterns of contact for functional discrimination of physical features of objects, such as compliance and texture. The proprioceptive afferents inform movement and position of hand digits during grasping, which leads to the perception of object size. Their efficient fusion allows humans to interpret complex object shapes (Jones and Lederman, 2006). The absence of these sensory afferents in amputees limits their ability of functional perception using commercial prosthetic hands (Johansson and Flanagan, 2009). Thus, they often rely heavily on visual information for prosthetic control (Biddiss and Chau, 2007; Engdahl et al., 2015). Although various techniques of invasive or non-invasive somatotopic sensory feedback have been developed in the recent decade to convey tactile information for forearm amputees (Bensmaia et al., 2020; Weber et al., 2020; Mendez et al., 2021), the lack of proprioceptive information of prosthetic finger movements makes it difficult for subjects to discriminate multiple features of objects, such as shape.

Various invasive or non-invasive techniques have been widely developed to elicit localized sensations referred to the missing hand (Lawrence et al., 2004; Polasek et al., 2009; Boretius et al., 2010; Sharma et al., 2011; Antfolk et al., 2012; Ortiz-Catalan et al., 2014; Tan et al., 2014; Chai et al., 2015; D'Anna et al., 2017; Shin et al., 2018; Marasco et al., 2021; Zhang et al., 2022b). The restored contact tactile information allows forearm amputees to perceive the intensity of contact force for discrimination of object compliance (Raspopovic et al., 2014; D'Anna et al., 2017; George et al., 2019; Valle et al., 2020; Vargas et al., 2021), while it is also possible to identify simple object shapes or locations via contact timing difference of different parts of hand after training (Raspopovic et al., 2014; D'Anna et al., 2017; Valle et al., 2020). One technique of non-invasive somatotopic tactile sensory feedback developed leverages transcutaneous electrical nerve stimulation (TENS) on the specific projected finger map (PFM) area at the stump (Chai et al., 2015). The induced finger-specific sensation, referred to as evoked tactile sensation (ETS), is conveyed to amputees via a direct afferent pathway from periphery receptors in the stump skin to the primary somatosensory cortex (SI) (Hao et al., 2020). The activation of sensory nerves and mechanoreceptors at the stump PFM elicits rich sensory percepts for robust encoding of multiple modalities of sensory information (Li et al., 2021; Zhang et al., 2022b). This explains that ETS can be interpreted and utilized intuitively by amputees for perceiving finger-specific contact or force (Hao et al., 2020; Zhang et al., 2022a). ETS shows a high spatial resolution for perception of individual fingers, which leads to the feasibility of sensory coding for up to five fingers (Chai et al., 2015; Hao et al., 2020). The information on contact pattern and force intensity via ETS feedback enables forearm amputees to identify object length or compliance (Zhang et al., 2022a). However, it conveys only tactile information that may be limited for complex object profiling.

Stable proprioception remains difficult to elicit by activating directly the sensory afferent pathway. Proprioception is mediated by sensory fibers innervating muscle spindles and tendon organs (Proske and Gandevia, 2012) or receptors around the joint (Edin and Abbs, 1991; Edin and Johansson, 1995; Collins and Prochazka, 1996). The lack of this information in prosthetic hands forces amputees to rely on visual feedback to control prosthetic hand motion (Atkins et al., 1996; Biddiss and Chau, 2007). Proprioceptive and kinesthetic sensations are elicited in a few invasive feedback methods (Dhillon and Horch, 2005; Horch et al., 2011; Tan et al., 2014; George et al., 2019; Segil et al., 2020). However, the limited modulation range for proprioception makes it difficult to encode the spatiotemporal proprioceptive information (Segil et al., 2020). Sensory substitution is extensively utilized to restore proprioception (Schiefer et al., 2018; Chai et al., 2019; D'Anna et al., 2019; Marasco et al., 2021; Zhang et al., 2022a). The combination of somatotopic tactile and substitute proprioceptive feedback is demonstrated to be a feasible way to expand the information bandwidth of a single afferent stream (Schiefer et al., 2018; D'Anna et al., 2019; Marasco et al., 2021; Zhang et al., 2022a). Our preliminary study also shows the possibility of integrating ETS tactile and substitute electrotactile feedback for the identification of single physical property (Zhang et al., 2022a). Combining tactile and proprioceptive information gives the feasibility for multi-dimensional discrimination in object profiling. However, simultaneous surface electrical stimulation to elicit ETS and electrotactile sensation may cause a mutual interference in communicating different sensory information clearly and in discriminating multiple features of objects accurately.

In this study, we hypothesize that dual-modality sensory information from somatotopic tactile feedback with substitute aperture feedback could be fused and interpreted by amputee subjects for multi-dimensional discrimination. We investigated the functional benefits of fusing the two steams of sensory information for perception and discrimination of object length and size. Specifically, the mutual interference effect of ETS and electrotactile stimulation was also examined. The natural, finger-specific ETS stimulation and substitute electrotactile stimulation were integrated to inform amputees of the contact pattern of five fingers and the hand aperture. This two-modality sensory feedback was incorporated into a commercial prosthetic hand to achieve closed-loop control and profile perception. To evaluate the mutual interference of somatotopic ETS and proprioceptive substitution, the task of length and size identification of objects was conducted separately or in combination. The ETS feedback in discriminating object length was robust with or without additional substitute electrotactile stimulation. The interference of ETS stimulation on substitute sensory perception was evaluated with stimulation sites at either the forearm or upper arm. The task of combined identification of length and size was then conducted to demonstrate the efficacy of fusing two afferent information streams. The findings confirmed the hypothesis for combining the non-invasive somatotopic tactile information with substitutive aperture feedback to improve the sensory capabilities of hand prostheses. Preliminary work was presented in a conference paper elsewhere (Li et al., 2021).

## 2 Materials and methods

### 2.1 Subjects

Two transradial amputees were recruited to participate in this study. Both subjects (S1, male, 54 years old; S2, male, 66 years old) were with left wrist disarticulation. All the experimental protocols were approved by the Institutional Review Board for Human Research Protections, Shanghai Jiao Tong University. Subjects were informed about all experimental procedures and signed the consent form before joining the study.

### 2.2 Experimental setup

**Figure 1:**
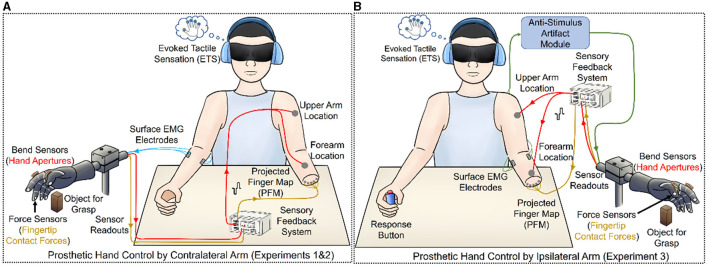
Experiment setup and the illustration of an amputee using closed-loop control of a prosthetic hand for grasping and identifying different objects via non-invasive sensory feedback. The subject was blindfolded and wore noise-canceling headphones. The sensory feedback signals were obtained by force and bend sensors in the prosthetic hand. The somatotopic ETS feedback was utilized to provide intuitive contact information with TENS at the PFM of stump skin, while the substitute aperture feedback located on the upper arm or forearm supplied the hand aperture information. **(A)** In Experiments 1 and 2, the contralateral right hand was used for grasp control (Blue Line). **(B)** In Experiment 3, the ipsilateral left hand operated grasp control and perceived the sensory information with the anti-stimulus artifact module. A response button was pressed after reporting the identification to record the response time.

Five force sensors (FlexiForce A201, Tekscan Inc, the United States) were placed on the fingertips of the prosthetic hand to collect the contact force. Two flexible bend sensors (Spectra Symbol, Salt Lake City, UT) were fixed along the thumb and index finger for measuring hand aperture. The hand aperture distance between the fingertips of thumb and index was calculated by the difference in voltage outputs of the two flexible sensors. A real-time non-invasive multi-channel stimulation pattern generator (Figure 1) was costume designed and constructed for this study. It could sample five channels of contact force signals and two channels of hand aperture signals at the frequency of 100 Hz, and deliver six channels of stimulation current pulses to corresponding stimulation electrodes. Either non-woven fabric circular electrodes (Yancheng Dalun Medical Equipment Co. Ltd, China) with 1 cm diameter were used in this study. Non-woven fabric reference electrode (with 5 cm diameter) of each channel was placed near the olecranon of elbow.

Figure 1 illustrates the experimental setup, in which the subject was seated at a table with eyes blinded and ears blacked with noise-canceling headphones. These setups were applied to block visual feedback and other incidental feedback, including auditory clue, transmitted by motor rotations. Two surface electromyographic (sEMG) sensors were placed on the wrist extensor and flexor carpi radialis to capture sEMG signals. In Experiments 1 and 2, the sEMG signals from the contralateral right arm were used to control the opening and closing of the Kesheng prosthetic right hand (Shanghai Kesheng Prostheses Co. Ltd, China) directly (Figure 1A). In Experiment 3, to further improve the integrated level of bidirectional prosthetic hand, the prosthetic hand (Otto Bock HealthCare, Germany) was changed into the ipsilateral control/sensing. The EMG signals of ipsilateral wrist flexor and extensor passed the anti-artifact model to remove the stimulation artifacts in real time (Yu et al., 2022) (Figure 1B).

### 2.3 Sensory feedback and coding strategy

In the somatotopic ETS feedback, the PFM was identified by pressing surface skin regions of the residual stump (Zhang et al., 2022b). The stimulation sites were fixed as the most sensitive points of five finger-specific PFM for tactile feedback of five fingers. For substitute aperture feedback, the stimulation site was set on the skin of the forearm or upper arm on the ipsilateral side of the amputation, which was both 10 cm away from the elbow and shoulder (Li et al., 2021).

The stimulation pulses of each channel were biphasic, charge-balanced, cathodic-first, rectangular pulses trains. An inter-pulse delay of 10 μs was present between the negative and the positive pulses (Szeto and Saunders, 1982). The pulse amplitude (PA), pulse width (PW) and pulse frequency (PF) of the current can be adjusted through the host computer. Since the buzz sensation was shown to have a wide pulse width modulation range and high sensitivity (Zhang et al., 2022b), it was chosen to encode sensory information.

For each stimulation site, PF was fixed at 50 Hz throughout the experiments. PA values and PW ranges were customized for each subject based on our previous protocols (Yang et al., 2020). PA increased from 1 mA with the step of 0.5 mA with fixed PF and PW (200 μs). The value that the buzz sensation could be perceived was identified as the fixed PA for this stimulation site. Then PW was increased from 10 to 600 μs with a step of 20–60 μs to find each *PW*_*up*_ and *PW*_*low*_, the maximum and minimum values corresponding to the buzz sensation.

**Figure 2:**
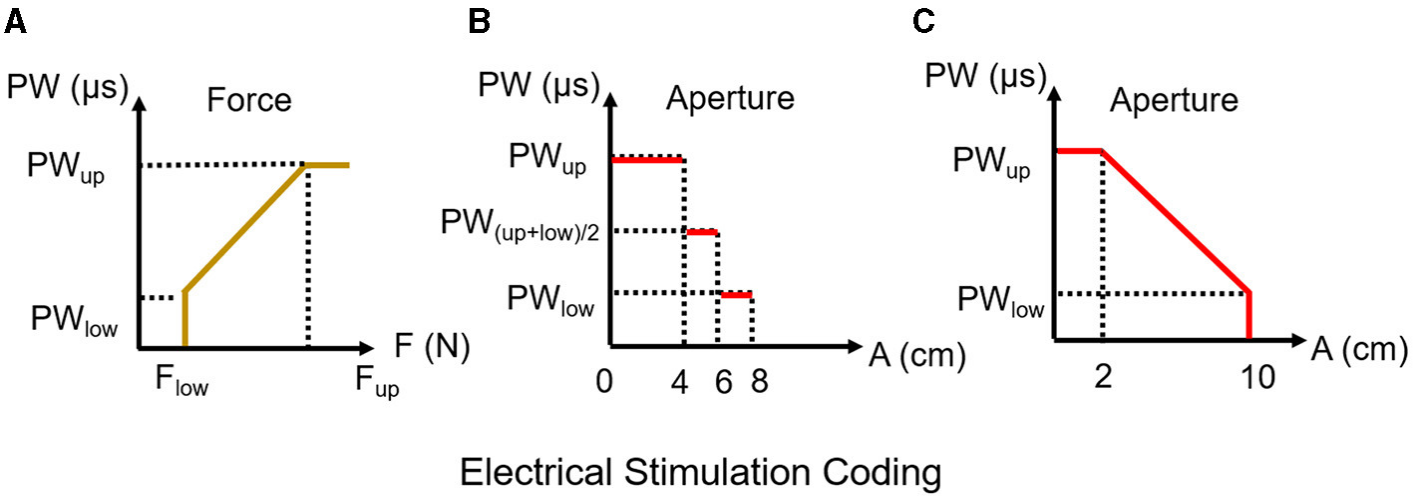
The coding strategy of contact force and hand aperture. **(A)** The linear coding relationship between fingertip force and stimulus PW output, which was shown in Equation (1). PW_up_ and PW_low_ are the maximum and minimum pulse width thresholds, respectively. F_up_ is the maximum contact force during grasping for each finger, while F_low_ is the minimum force readout before grasping. **(B, C)** Show the staircase coding in Equation (2) and linear coding in Equation (3) for hand aperture.

The coding strategy relationships between the sensor readouts and electrical stimulus are shown in Figure 2. The maximum and minimum force readouts of each finger were recorded and used as the upper and lower limits of the contact force, *F*_*up*_ and *F*_*low*_, respectively. Contact forces were mapped to PWs (Figure 2A) according to Equation (1),


(1) $$PW=\frac{\;(PW_{up}-PW_{low})}{F_{up}-F_{low}}*\left(F-F_{low}\right)+PW_{low}$$

where *F* is the contact force between the prosthetic fingertip and the object. *F*_*up*_ is set as 15 N and *F*_*low*_ is 0 N.

The hand aperture (opening distance) was mapped to PW (Figure 2B) by segments according to Equation (2),


(2) $$PW=\{\begin{array}{c} PW_{up},\;When\;0<A\leq A_{small}\\ \frac{\left(PW_{up}+PW_{low}\right)}{2},\;When\;A_{small}<A\leq A_{med}\;\\ PW_{low},\;When\;A_{med}<A\leq A_{large}\\ 0\;,\;When\;A>A_{large} \end{array}$$

where *A* is the distance between the thumb and index finger, *A*_*small*_, *A*_*med*_, and *A*_*large*_ are equal to 4, 6, and 8 cm, respectively.

In particular, the complexity in Experiment 3 was adjusted with a design that was more relevant to activities of daily living, allowing the amputee to identify the opening/closing distance of the object electrical stimulus coding from the segments Equation (2) way to a linear coding Equation (3), by linear coding the amputee to judge the object as “sm” (small) or “lg” (large) (Figure 2C). Since the surface electrical stimulation for the same object was consistent, subjects could utilize same strategy to identify object size in different coding strategy.


(3) $$PW=\frac{\;(PW_{up}-PW_{low})}{P_{up}-P_{low}}*\left(A_{\mathrm{lg}}-A\right)+PW_{low}$$

where *PW* changes linearly with *A* when *A* is between 2 and 10 cm (*A*_lg_).

### 2.4 Experiment design

#### 2.4.1 Experiment 1: evaluation of length identification

**Figure 3:**
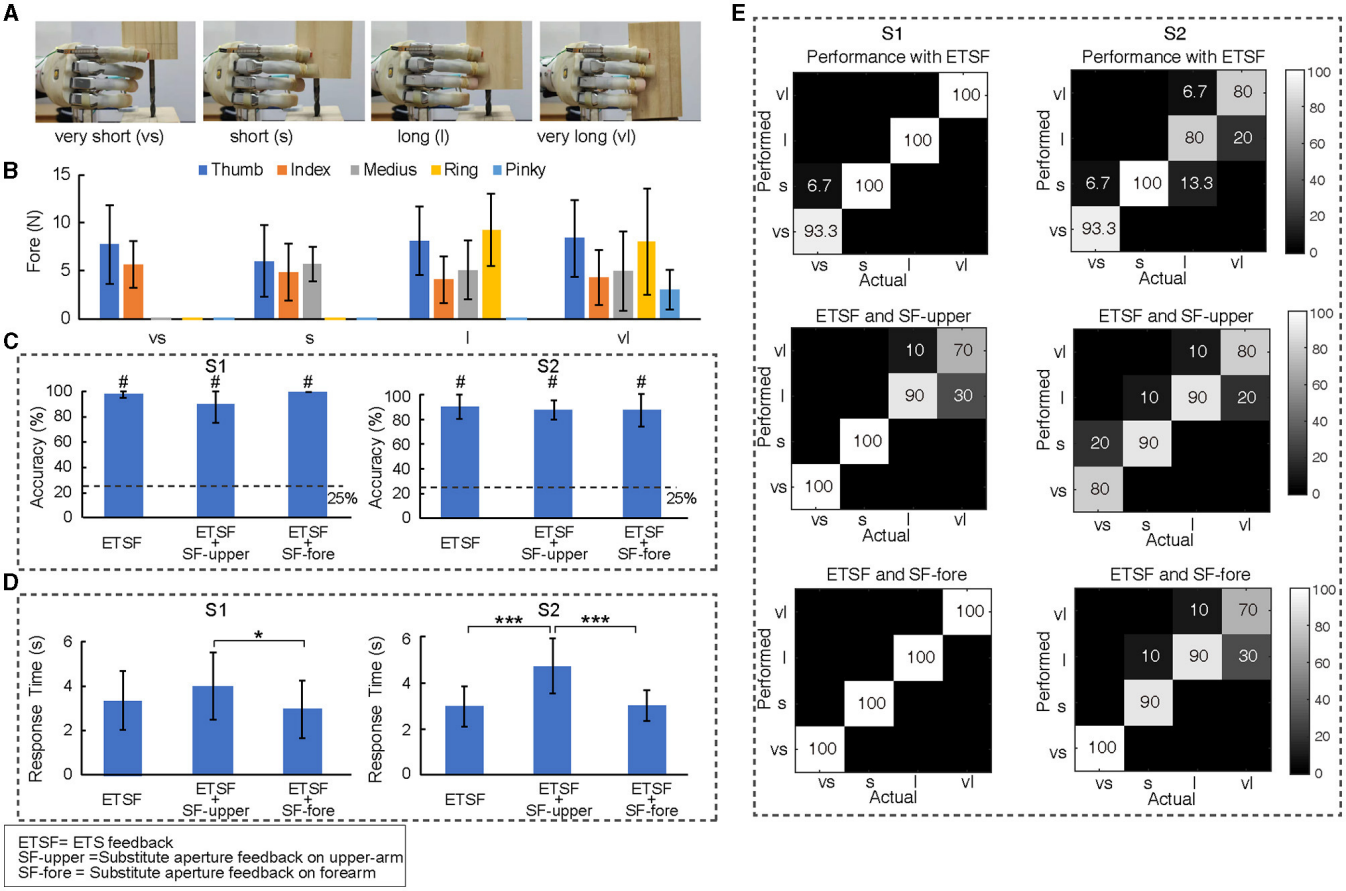
Identification of object length. **(A)** Four objects with different lengths and same size were used in the length identification task and their labels. **(B)** The pattern of contact forces of five fingers from both subjects when grasping objects of four different lengths under all conditions. **(C, D)** The accuracy and response time with respect to three different feedback conditions. “#” identifies the level that is significantly different from the chance level. **(E)** The performance in the confusion matrix for both subjects identifying each object. *p < 0.05, ***p < 0.001).

In this task, subjects were asked to control the prosthetic hand to grasp the object using power grip. With this grip, all fingers were bent toward the object. When the finger touched the object, its contact force information was registered and conveyed back to the subject via ETS stimulation. If not, he finger continued to bend down until maximal flexion with no contact force. Then, they would identify the length of an object, chosen from four object lengths [6 cm (very short), 8 cm (short), 10 cm (long), and 12 cm (very long)] (Figure 3A) and reported orally. Different object lengths led to different numbers of contact fingers that would convey to subjects (Figure 3B). The ETS feedback was utilized to supply contact information, while the substitute subject on the ipsilateral left forearm (SF-fore) or left upper arm (SF-upper) was supplied to evaluate its effect on the efficacy of ETS feedback. The grasp of the prosthetic hand was controlled by the amputee's contralateral right hand. Each object appeared 15 times with ETS feedback and 10 times with other conditions randomly in 5 sessions.

#### 2.4.2 Experiment 2: evaluation of hand aperture identification

**Figure 4:**
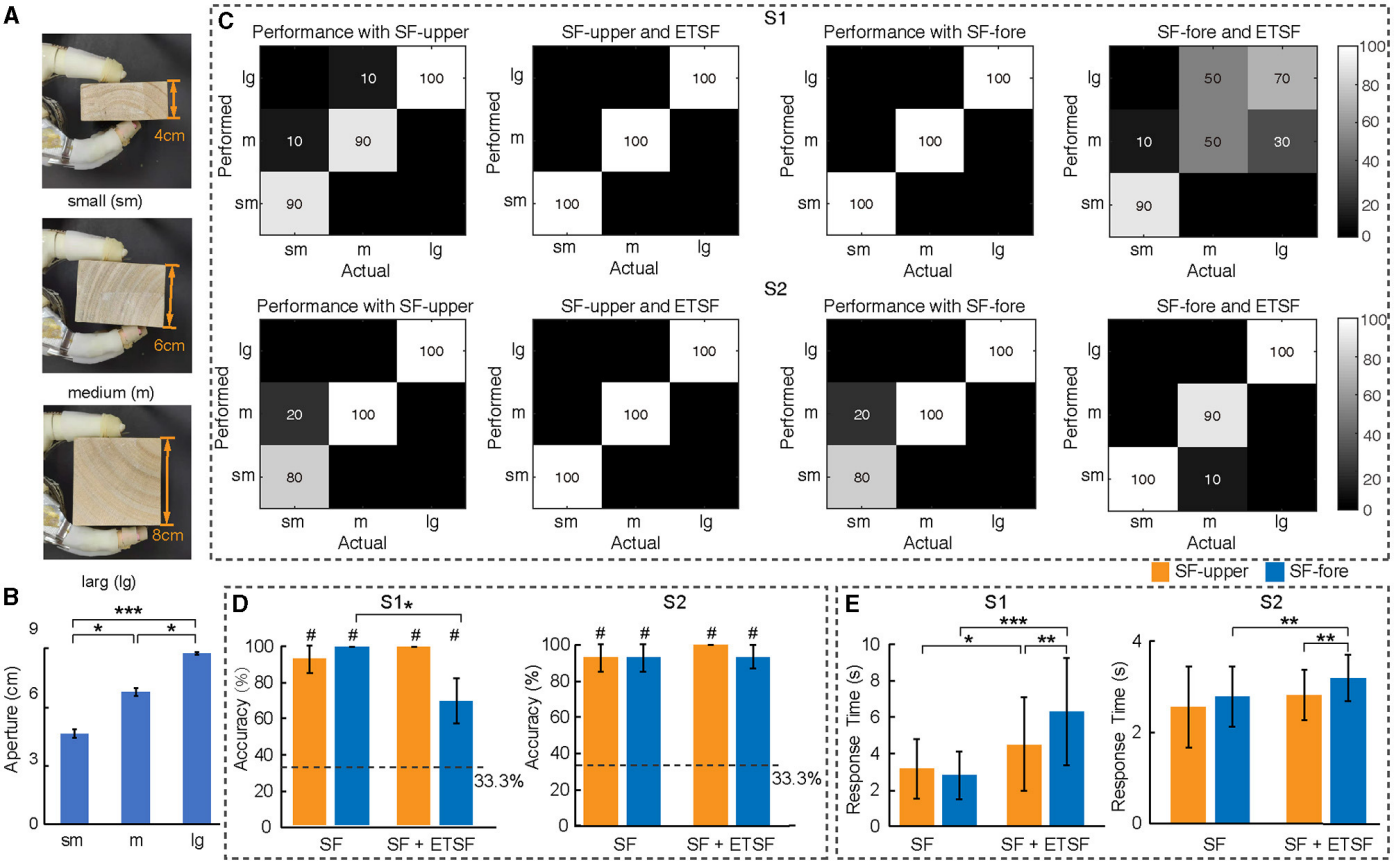
Identification of object size. **(A)** Objects with three sizes at the same length used in size identification experiments. **(B)** The hand aperture information when S1 and S2 grasped objects of three different sizes under all conditions, i.e., substitute aperture feedback on upper-arm (SF-upper) and forearm (SF-fore), as well as with additional ETS feedback (ETSF) simultaneously. **(C, D)** The accuracy of size classification under four feedback conditions in the form of confusion matrix and overall performance, respectively. “#” identifies the level that is statistically different from the chance level. **(E)** The response time for S1 and S2 under four feedback conditions. *p < 0.05, **p < 0.01, and ***p < 0.001.

During the size identification, three blocks of equally spaced sizes (4, 6, and 8 cm for small, medium and large blocks) were used (Figure 4A). Different sizes of objects during grasping were recorded (Figure 4B). With SF-upper/SF-fore, the subject was asked to grasp the object with power grip and report its size with or without the additional ETS feedback (ETSF). The grasp of the prosthetic hand was controlled by the amputee's contralateral right hand. Each object was randomly presented 15 times on each condition in five sessions.

#### 2.4.3 Experiment 3: evaluation of length and hand aperture identification

**Figure 5:**
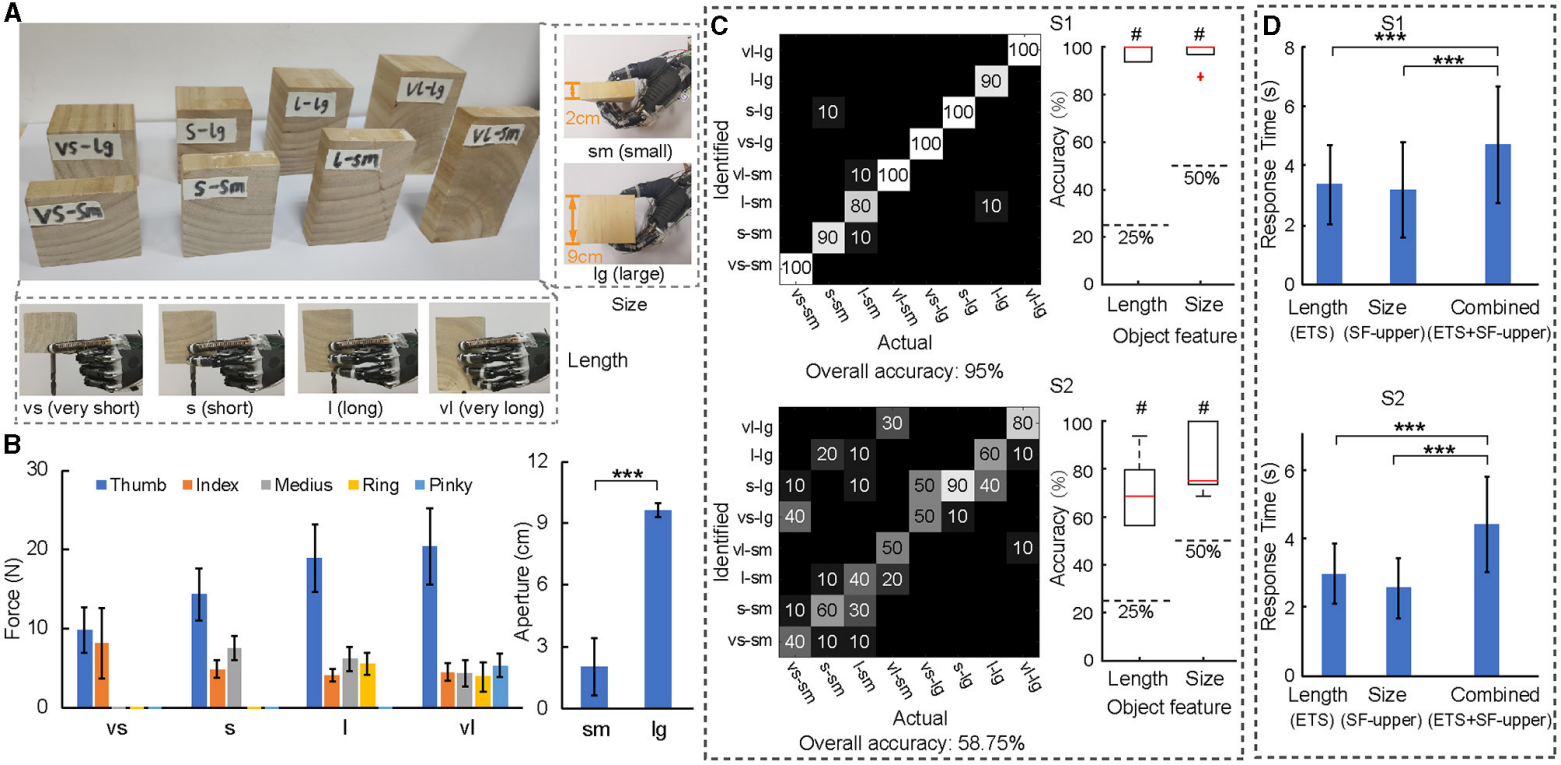
Identification of object length and size. **(A)** Eight wooden blocks with four different lengths and two different sizes in this experiment session. **(B)** The overall contact force of five fingers and hand aperture information when grasping different objects by all subjects. **(C)** Performance during the task with ETS feedback and substitute aperture feedback on the upper-arm for S1 and S2 in the form of a confusion matrix **(left)** and boxplot identifying size/length **(right)**. “#” identifies the level that is statistically different with the chance level. **(D)** Response time to identify each object in single size identification with ETS feedback (ETSF), single length identification with substitute aperture feedback on upper-arm (SF-upper), and the combined size and length identification task. “***”*p* < 0.001.

In the combined size and length identification task, objects with the same lengths as the length identification task and two sizes [2 cm (small) and 9 cm (large) in width] were used (Figure 5A). Both contact finger and aperture information were conveyed to subjects by ETS and substitute aperture feedback on upper-arm (Figure 5B). To further test subject's ability of ipsilateral closed-loop control for prosthetic hand, a Bebionic hand with the same myoelectric control strategy was utilized in Experiment 3. The change to ipsilateral side may influence the task complexity for amputee subjects to control the bidirectional prosthetic hand. Thus, before Experiment 3, each subject was given a training session of 5–10 min to familiarize the ipsilateral control and sensing. The subject was asked to answer the length and size of the object to be grasped and pressed the response button the simultaneous time under the control of the prosthetic hand of the amputated side. This experiment consisted of five sessions and each object was presented 10 times randomly.

Identification accuracy and response time were recorded to evaluate the subjects' ability to perceive multiple fingers. Response time was defined as the interval between the moment when the contact force rose and when the subject reported the answer in Experiments 1 and 2, or when he pressed the response button in Experiment 3. Confusion matrixes were constructed for block size identification experiments to show the actual and perceived results by the subjects.

### 2.5 Statistical analysis

All data were analyzed using IBM SPSS Statistics version 26.0 (SPSS Inc., Chicago, IL, USA, 2019). The one-sample Kolmogorov-Smirnov test was used to test if the dataset was normally distributed. Since none dataset passed the test, Kruskal-Wallis test (K-W test) was used to determine whether there was a significant difference in accuracy, response time and aperture. The identification of both subjects was compared against chance level with the Binominal test.

## 3 Results

### 3.1 The ETS stimulation for multiple digits feedback

The role of multi-digit ETS feedback in the identification of object dimension was evaluated first with objects of four different lengths (Figure 3A). Without visual and auditory feedbacks, length identification of objects resulted primarily from distinguishing the fingers contacting with the object (Figure 3B). Evaluation of efficacy of ETS feedback was conducted under three conditions: (1) ETS feedback only (ETSF), ETS feedback with substitute stimulation on upper-arm (SF-upper) (2) or forearm (SF-fore) (3). Results showed first that ETS feedback alone allowed both subjects to identify object length with high accuracy (98.3% for S1 and 90.0% for S2) (Figure 3C). This indicated that the ETS feedback provided sufficient information for length classification through the pattern of contacting fingers. Second, both subjects could maintain a high accuracy of length identification with additional substitute stimulation on upper-arm (90.0% for S1 and 87.5% for S2) or forearm (100% for S1 and 87.5% for S2) (Figure 3C). Although some confusion occurred between “long” and “very long” lengths (Figure 3E), the overall performance was all above the chance level (Figure 3C). These results suggested that both subjects acquired the ability of robust perception of the basic pattern of contacting fingers via ETS feedback despite the additional substitute stimulation (Figure 3B). Response time of identification appeared to vary with subjects and testing conditions, but both subjects could in general interpret basic information provided in ETS feedback on the order of 3–5 (s) (Figure 3D). This finding further supported the notion that it is possible to combine ETS feedback with substitute stimulation to inform more sensory information.

### 3.2 Substitute stimulation for hand aperture (opening) feedback

The role of substitute stimulation on the upper-arm or forearm to inform object size was evaluated with an aim to combine with the ETS feedback. Test results showed significantly different levels of stimulation relating to hand apertures (Figure 4B). Under substitute stimulation only, both subjects could identify object size with high accuracy on either upper-arm stimulation (93.3% for S1 and 93.3% for S2) or forearm stimulation (100% for S1 and 93.3% for S2) (Figures 4C, D). Distal ETS stimulation in both subjects affected size perception with substitute simulation on forearm more notably than that on upper-arm (Figure 4C). The performance under four different feedback conditions was all above the chance level (Figure 4D). Response time appeared to vary with subjects and ETS stimulation (Figure 4E). These results suggested that upper-arm substitute feedback might better isolate perception interference from ETS. Nevertheless, substitute feedback conveyed sufficient information regarding the size dimension of object via hand aperture/opening. Therefore, it might be beneficial to fuse the ETS feedback and substitute feedback at upper-arm stimulation to provide multi-modality sensory information.

### 3.3 Fusion of ETS and substitute stimulation for multi-modality sensory feedback

A more challenging task of recognizing object size and length simultaneously was conducted to understand the efficacy of perceiving multi-modality sensory information via ETS and aperture feedbacks on upper-arm. The ETS pattern of contact digits and hand aperture information was presented to the subject for identification (Figure 5B). The overall accuracy for length and size identification was 95% for S1 and 58.75% for S2, all of which were above the chance level (Figure 5C). The feature of only size or length was all identified above chance levels (Figure 5C). But S2 had a much higher rate of success in recognizing size than length (Figure 5C). Results indicated that information provided by the two sensory feedbacks could be integrated for size and length perception by the two subjects. In both cases, the response time in the combined size and length identification was longer than that in a single size or length identification (Figure 5D). This showed that task complexity added a greater burden to the cognitive integration and processing of sensory information. This test established that ETS-based multi-digit tactile sensory feedback could be combined with substitute aperture feedback to provide two-dimensional information about objects, and the information could be integrated and processed by both subjects in distinguishing object size and length.

## 4 Discussion

The sensory information bandwidth of a single sensory feedback approach is limited for object profiling. To provide multiple sensory information, the combination of somatotopic and substitute sensory feedback approaches has been demonstrated to be effective for the identification of various physical properties (Schiefer et al., 2018; D'Anna et al., 2019; Vargas et al., 2021; Zhang et al., 2022a). In our approach with ETS-based somatotopic sensory feedback, previous studies showed the ability to restore the finger-specific tactile sensation via a naturally regenerated afferent pathway (Hao et al., 2020; Zhang et al., 2022a). This study was to explore the neurocognitive ability of amputees to fuse two different streams of somatotopic tactile and substitute aperture information. Results confirmed the robustness of somatotopic ETS and substitute aperture feedbacks for dimensional perception. The study demonstrated the feasibility to combine two different information streams by amputee subjects for effective functional object profiling. Results also suggested the optimal location for substitute aperture stimulation in the upper arm of amputees.

Somatotopic ETS feedback displayed robust communication of tactile information against distortion from distant stimulation on the ipsilateral arm for substitute aperture feedback. The evoked afferents can be perceived by amputees as occurring in the lost digits in a natural, intuitive and direct way (Hao et al., 2020). The finger-specific sensation allowed subjects to identify the object length effectively (Figure 3B). Both subjects achieved high success rates by relying on discriminating the contact pattern of fingers provided by ETS (98.3% for S1 and 90.0% for S2). The performance was comparable to those reported in the literature in similar tasks that leveraged timing differences in contact location information with invasive peripheral nerve stimulation at 97.3% (Raspopovic et al., 2014) or 95% (Segil et al., 2020), and proximal nerve stimulation at 84.3% (D'Anna et al., 2017) (Table 1). Among these techniques, ETS-based feedback is one of the few methods (Segil et al., 2020) capable of both individually and simultaneously conveying tactile information up to five fingers. There was no difference in accuracy with or without substitute feedback on forearm and upper arm (*p* > 0.05, Figure 3C). This demonstrated that the ability of both amputee subjects to interpret the somatotopic tactile information were preserved even with concurrent substitute electrotactile stimulation (Figure 3C). The impact of additional sensory stimulation on the efficacy of ETS feedback mainly showed in the response time of identification (Figure 3D). Both subjects needed less identification time when the sensory substitution was closer to the PFM area (*p* < 0.05). It may be related to the cognitive process of perceiving two sources of information separated in different locations in the arm. The confusion of identification for S2 mainly occurred between “long” and “very long” objects (Figure 3E). It may be due to that the pinky finger had relatively low force intensity compared to other fingers, which may be related to the relative position of object and prosthetic hand. This task demonstrates that amputee subjects could focus and interpret the intuitive finger-specific tactile information even with two simultaneous afferent information streams. Since the ETS feedback delivers a natural digit-specific afferent stream to the proprio-somatotopic hand areas in SI (Hao et al., 2020), it does not interfere with the substitutive afferent delivered to the upper arm area in SI. This implies that the somatotopic separated sensory afferents can be more clearly represented in SI and more robustly interpreted in the fusion of sensory information (D'Anna et al., 2019; Valle et al., 2020).

**Table 1:** Comparison of literatures in object feature identification via multiple-finger information.

**Article**	**Raspopovic et al. (2014)**	**Segil et al. (2020)**	**D'Anna et al. (2017)**	**Zhang et al. (this one)**
Technique	Transversal intrafascicular multichannel electrodes	Flat interface nerve electrodes	Proximal nerve stimulation	Transcutaneous electrical nerve stimulation
Task	Object position identification (three types)	Hand posture identification (four types)	Object location identification (three types)	Object length identification (four types)
Feedback information	Index and little fingers	All five fingers	Ulnar region and median region	All five fingers
Accuracy	97.3%	95%	84.3%	94.15%

The accuracy shown in this table is average number.

Different from robust ETS-based feedback, the substitute sensory feedback could be disturbed by distal ETS stimulation. For size identification with upper-arm stimulation, our performance (93.3% for S1 and 93.3% for S2) was comparable to that of peripheral nerve stimulation at 96.9% (George et al., 2019), or substitute stimulation at 78% (D'Anna et al., 2019) and 84% (Su et al., 2022). Meanwhile, the average response time of our subjects was 4.01 (s) in S1 and 4.72 (s) in S2, much shorter than 8.94 (s) in the previous study (George et al., 2019) (Table 2). The identification accuracy of S1 was significantly impacted by ETS stimulation when the substitute aperture feedback was placed on the forearm (*p* < 0.05, Figure 4D). In this feedback scenario, both subjects required more response time with additional ETS stimulation (*p* < 0.01, Figure 4E), where upper arm stimulation required less time than forearm stimulation (*p* < 0.01). This space-dependent effect implied that there may be an electric field interference to substitute perception. Considering the robustness of ETS feedback and the minimal crosstalk in both electric field and cortical SI representation, placing the sensory substitution stimulation on the upper arm may be a preferred choice for further integration of multi-modality sensory feedback. It suggests a guideline for choosing the location of sensory substitution in the integration of an electrotactile feedback with the ETS feedback in the ipsilateral arm of amputees.

**Table 2:** Comparison of literatures in object size identification.

**Article**	**George et al. (2019)**	**D'Anna et al. (2019)**	**Su et al. (2022)**	**Zhang et al. (this one)**
Technique	Utah slanted electrode array	Transversal intrafascicular multichannel electrodes	Surface electrical stimulation	Transcutaneous electrical nerve stimulation
Task	Object size identification (two types)	Object size identification (four types)	Object size identification (three types)	Object size identification (three types)
Feedback information	Index fingers	Remapped proprioception	Remapped proprioception	All five fingers
Accuracy	96.9%	78%	84%	93.3%
Response time	8.94 s	–	–	4.365 s

The accuracy and response time shown in this table is average number.

The ability to interpret two different information streams relating to the ETS in digits and the aperture of hand was further evaluated. In combined identification tasks with length information via ETS and size information via substitute stimulation, both length and size of objects could be identified simultaneously with an accuracy of 95% by S1 and 58.75% by S2, all were above the chance level (*p* < 0.05, Figure 5C). Our previous study showed that this hybrid multi-modality feedback enabled the forearm subject to identify object compliance with the accuracy of 100 % (Zhang et al., 2022a). These results illustrated that amputee subjects could not only fuse two different afferent sensory information to confirm a single object feature, but also flexibly utilize them for the identification of multiple physical properties. It also indicated the feasibility of hybrid ETS and substitute aperture feedback, which allowed effective identification of object length, size, and compliance. The information dimension conveyed by this non-invasive approach was comparable to that of previous studies (Schiefer et al., 2018; D'Anna et al., 2019; Vargas et al., 2021). Both subjects required more time in the identification of combined features than single size or length (*p* < 0.001, Figure 5D), which indicated that the cognitive process may be related to the complexity of the task and the channels of information being processed simultaneously (Freides, 1974; Ernst and Bülthoff, 2004; Antfolk et al., 2013), as well as the prior information available for the integration (Klatzky et al., 1985; Ernst and Bülthoff, 2004). The fusion of ETS and substitute aperture feedbacks for object profiling requires a higher level of ability to integrate received information and to make an optimal judgment based on perception (Ernst and Banks, 2002). Since these two sensory afferents interact with each other subconsciously, the reliability and variance of sensory fusion may require learning to obtain optimal discrimination of integrated information (Ernst and Bülthoff, 2004; Dayan and Daw, 2008; Štrbac et al., 2017). However, the effects of learning await further investigation in future studies.

There were two limitations in this study. Although the sensory fusion study was tested only in two amputee subjects, the performance trends of both subjects were consistent, demonstrating that the neurocognitive ability of subjects is capable of processing two modalities of simultaneous sensory information effectively. In future studies, this multi-modality sensory feedback will be evaluated in a larger population of forearm amputee subjects to demonstrate functional improvements in tasks more relevant to daily living and working. Second, two different commercial prosthetic hands were used in the combined identification task and single feature identification using the bidirectional prosthetic hand. This was a necessary step to upgrade from contralateral control/sensing to more integrated ipsilateral control/sensing, and test the effectiveness of ipsilateral bi-directional communication. With some training, both subjects were able to overcome the change brought about by switching from contralateral control/sensing to ipsilateral communication. However, the control mode and experimental paradigm for both prosthetic hands remained the same in all tasks, which ensured consistency in results.

This study investigated and demonstrated the neurocognitive ability of amputees to combine dual-modality of sensory information based on the somatotopic ETS and the substitute stimulation. The ETS feedback showed a robust ability to convey finger-specific information in the presence of additional sensory stimulation. The farther away the aperture stimulation location from the stump site of ETS stimulation, the less interference to aperture perception from ETS stimulation. Thus, the substitute aperture feedback in upper arm stimulation was the preferred way to incorporate with the ETS feedback in amputees. Test results confirmed that the hybrid sensory feedback enabled both subjects to identify the combined size and length of grasped objects effectively. This study reveals the robustness of non-invasive ETS to restore somatotopic sensation and illustrates the cognitive ability of amputees to fuse different sensory information for effective functional discrimination. This study established the feasibility of sensory fusion in amputees to combine tactile and proprioceptive information through non-invasive techniques of sensory feedback for improving prosthesis functionality.

## Data availability statement

The raw data supporting the conclusions of this article will be made available by the authors, without undue reservation.

## Ethics statement

The studies involving humans were approved by Institutional Review Board for Human Research Protections, Shanghai Jiao Tong University. The studies were conducted in accordance with the local legislation and institutional requirements. The participants provided their written informed consent to participate in this study. Written informed consent was obtained from the individual(s) for the publication of any potentially identifiable images or data included in this article.

## Author contributions

JZ: Data curation, Methodology, Validation, Writing—original draft, Writing—review & editing. C-HC: Methodology, Software, Writing—original draft. MH: Methodology, Supervision, Writing—original draft. YL: Writing—original draft. YY: Writing—original draft. NL: Writing—original draft, Writing—review & editing.
